# Three-Dimensional Laser Printing of Macro-Scale Glass Objects at a Micro-Scale Resolution

**DOI:** 10.3390/mi10090565

**Published:** 2019-08-26

**Authors:** Peng Wang, Wei Chu, Wenbo Li, Yuanxin Tan, Fang Liu, Min Wang, Jia Qi, Jintian Lin, Fangbo Zhang, Zhanshan Wang, Ya Cheng

**Affiliations:** 1 School of Physics Science and Engineering, Tongji University, Shanghai 200092, China; 2 State Key Laboratory of High Field Laser Physics, Shanghai Institute of Optics and Fine Mechanics, Chinese Academy of Sciences, Shanghai 201800, China; 3 University of Chinese Academy of Sciences, Beijing 100049, China; 4 XXL—The Extreme Optoelectromechanics Laboratory, School of Physics and Materials Science, East China Normal University, Shanghai 200241, China; 5 School of Physical Science and Technology, ShanghaiTech University, Shanghai 200031, China; 6 State Key Laboratory of Precision Spectroscopy, East China Normal University, Shanghai 200062, China; 7 Collaborative Innovation Center of Extreme Optics, Shanxi University, Taiyuan 030006, China

**Keywords:** ultrafast laser microfabrication, 3D glass printing, light-field manipulation

## Abstract

Three-dimensional (3D) printing has allowed for the production of geometrically complex 3D objects with extreme flexibility, which is currently undergoing rapid expansion in terms of materials, functionalities, as well as areas of application. When attempting to print 3D microstructures in glass, femtosecond laser-induced chemical etching (FLICE)—which is a subtractive 3D printing technique—has proved itself a powerful approach. Here, we demonstrate the fabrication of macro-scale 3D glass objects of large heights up to ~3.8 cm with an identical lateral and longitudinal feature size of ~20 μm. The remarkable accomplishment is achieved by revealing an unexplored regime in the interaction of ultrafast laser pulses with fused silica, which results in depth-insensitive focusing of the laser pulses inside fused silica.

## 1. Introduction

Thanks to the ultrashort pulse durations, ranging from femtosecond to tens of picoseconds, and the ultrahigh repetition rates, enabling the efficient and high throughput machining of materials, ultrafast lasers nowadays have been widely adopted in fabricating three-dimensional (3D) microstructures in various transparent materials [1,2,3,4,5,6,7]. In particular, ultrafast lasers have enabled the fabrication of geometrically complex 3D microstructures in glass for a variety of applications ranging from microfluidics and micro-optics to micromechanics [8,9,10]. Generally speaking, ultrafast laser pulses with sub-ps durations are considered more advantageous than the picosecond laser pulses in terms of the highest achievable spatial resolution as well as energy deposition efficiency, as the shorter the laser pulse durations, the less significant the thermal diffusion and the stronger the interaction of the laser pulses with the materials owing to the enhanced peak intensities [11]. For instance, when combining the focusing power of high numerical aperture (NA) lenses and the extreme sensitivity in the response of large bandgap glass materials to laser intensity, the femtosecond laser pulses have enabled the achievement of a nanoscale feature size in both surface and in-volume structuring of glass [12,13]. The facts seemingly encourage choosing only high-NA objectives for high-resolution 3D structuring of glass, owing to a rapid reduction of axial resolution with the decrease of NA. With the diffraction of light waves, the FWHM (full width at half maximum) of the size of the focal region is inversely proportional to the NA of the focal lens in the linear interaction regime [14], and the resolution can be further spoiled when the filamentation introduced by nonlinear self-focusing of the pulses is taken into account [15]. 

Unfortunately, the high-NA focal systems are inherent in short working distances. Thus, the depth of the focal position in the glass allowed by the high-NA lens is typically limited to a few millimeters, which is often desirable to be significantly extended. Previously, a simultaneous spatiotemporal focusing (SSTF) scheme was proposed to tackle this problem, which is able to maintain a reasonable axial resolution even with low-NA focal lenses [16,17]. In this scheme, the spectral components of the femtosecond pulses are separated in space before entering the objective lens. The spatial overlapping of different frequency components only occurs at the focus, leading to the shortest pulse duration and highest peak intensity. As a result, nonlinear self-focusing before the focus can be avoided, and the axial resolution of fabrication will be improved because the peak intensity will decrease rapidly when moving away from the geometric focal spot. Recently, this scheme has enabled 3D micro-printing of fine-featured objects in polymer with heights up to 1.3 cm [18,19]. Nevertheless, implementation of the SSTF scheme adds extra complexity and cost in femtosecond laser 3D micromachining. Here, we show an amazing and unexpected finding that a nearly spherical modification volume can be produced at arbitrary depths within fused silica with a low-NA focal lens by temporally chirping the femtosecond laser pulses into picosecond laser pulses. In other words, one can now achieve depth-insensitive-focusing using long working distance focal lenses regardless of the focal position within fused silica. This unique characteristic provides an opportunity to accomplish laser printing of macro-scale 3D objects in glass at a micro-scale feature size in an easy and flexible manner, which has never been achieved in femtosecond laser-induced chemical etching (FLICE) despite the great effort spent on it over the past two decades.

## 2. Materials and Methods 

**Figure 1:**
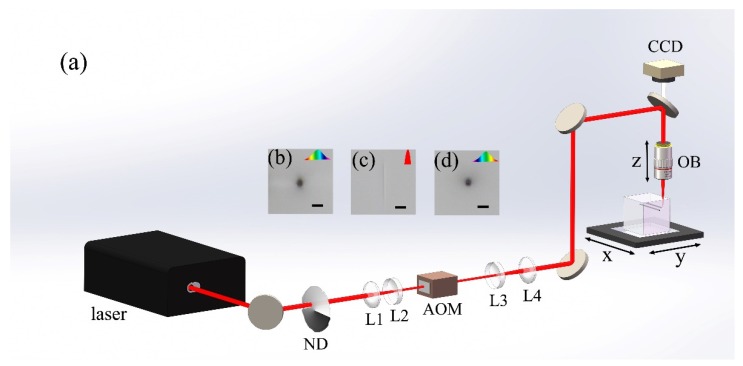
(**a**) Schematic of the experimental setup. ND, variable neutral density filter; L1 and L4, convex lens; L2 and L3, concave lens; AOM, acousto-optical modulators; CCD, charge-coupled device; OB, objective lens. Cross-sectional view of optical micrographs of lines inscribed in fused silica with (**b**) positively chirped 10 ps laser pulses, (**c**) transform-limited 190 fs laser pulses, and (**d**) negatively chirped 10 ps laser pulses. Scale bar: 25 μm.

The experimental setup is schematically illustrated in Figure 1. A Yb:KGW (Ytterbium doped Potassium-Gadolinium Tungstate) femtosecond laser (Pharos PH1-SP, Light Conversion) generated short pulses at a 1030 nm wavelength with the highest single pulse energy at 1 mJ. The pulse duration can be continuously tuned from 0.19 to 10 ps with either a positive or negative chirp. The repetition rate of the laser source can be adjusted between 1 kHz and 1 MHz. When performing the glass 3D micro-printing, the repetition rate was set to be 200 kHz, and the laser power was controlled using an attenuator. The laser beam was first reduced to 2 mm in diameter using a telescopic system, and then passed through an acousto-optical modulator (AOM). The AOM was triggered by a radio frequency signal from the controller of the transition stage to offer a fast shutter speed less than 100 μs. Afterwards, the laser beam was expanded using a beam expander and focused into the sample (i.e., a 55 mm-thick cube of fused silica) using a 5× objective lens (M Plan Apo NIR, Mitutoyo Corporation, Kawasaki, Japan ) with a numerical aperture (NA) of 0.14. The objective lens features a long working distance of 37.5 mm, enabling the fabrication of structures with heights up to 5 cm in fused silica. A 1D stage (ANT130-110-L-ZS, Aerotech Inc., Pittsburgh, PA, USA) was used to translate the objective lens along the Z direction to control the depth of the focus position in the glass. The glass sample was mounted on an XY motion stage (ABL15020WB and ABL15020, Aerotech Inc., Pittsburgh, PA, USA) which controlled the lateral motion of the sample with a scan speed up to 30 mm/s at a sub-500 nm positioning precision. Both translation stages were controlled by the machine controller (A3200, Aerotech Inc., Pittsburgh, PA, USA) and synchronized with the AOM. The combination of the high scan speed and the high positioning precision facilitated the rapid 3D micro-printing of large structures in the glass, as will be shown below.

The models used for 3D glass micro-printing were originally generated as stereolithography (STL) files. The STL files were firstly covered by a cuboid frame and then underwent a subtraction operation. Afterwards, the 3D models were sliced into horizontal planes with a fixed slice thickness. We scanned the laser focal spot in the sliced planes along the pre-designed paths layer by layer to produce the 3D structures. The scan process was performed from the bottom to the top of the glass. Typically, there are two scan strategies in the stereolithography fabrication: The raster scan and the contour scan. In the former strategy, the whole volume of the structure should be scanned; whereas for the later one, the laser focal spot only scans along the contour profile of the 3D structure. In our experiment, since the irradiated part of the glass should be removed after wet etching to get the structure, the raster scan had been chosen.

**Figure 2:**
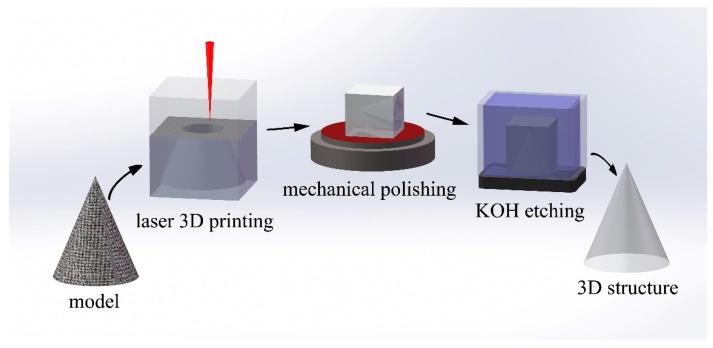
The four major steps in the 3D glass micro-printing: Digitalization of the 3D model (first panel), scanning of the laser beam along the pre-designed paths to selectively modify the areas surrounding the 3D objects (second panel), mechanical polishing (third panel), removal of the irradiated materials with chemical wet etching (fourth panel). The printed 3D structure is illustrated in the last panel.

After the fused silica samples were selectively irradiated by the focused laser pulses, the samples were polished to remove the outer areas as illustrated in the first three panels of Figure 2. The outer areas near the vertical sidewalls of the cubic sample could not be sufficiently modified by the laser irradiation because of the distortion of the focal spot caused by the air–glass interface at the sidewalls. The polished samples were then immersed in a wet-etching bath of potassium hydroxide (KOH) with a concentration of 10 mol/L at a temperature of 90 °C for tens of hours, as shown in the last two panels of Figure 2. For example, it took in total ~72 h (the etch rate was estimated to be 0.15 cm^3^/h) in the etching process to produce the 3.8 cm-tall Confucius sculpture, as will be shown below.

## 3. Results and Discussion

### 3.1. Depth-Insensitive Focusing in Fused Silica with Loosely-Focused Picosecond Laser Pulses

**Figure 3:**
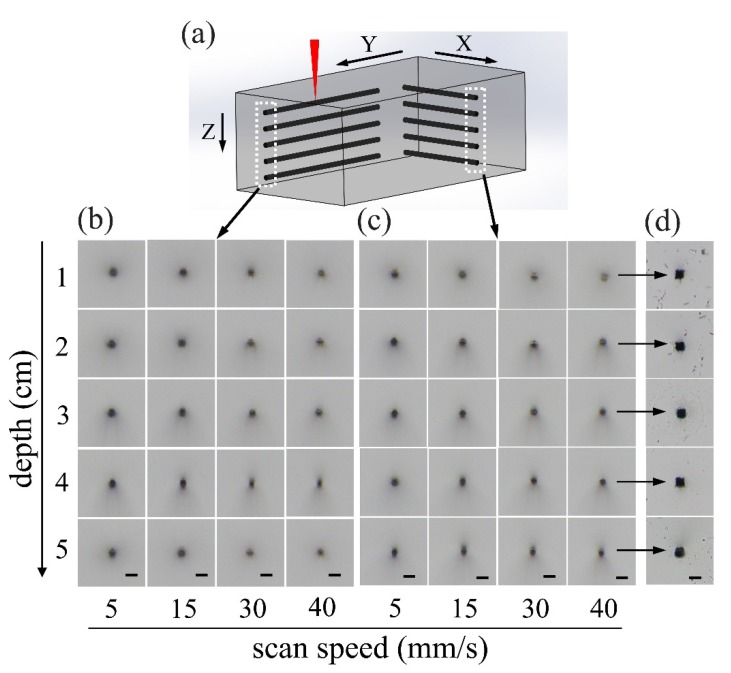
The fabrication feature size offered by loosely focusing the picosecond laser pulses into fused silica. (**a**) Schematic illustration of inscribing lines within a cube of fused silica along the X and Y direction. Cross-sectional optical micrographs of the lines written along the (**b**) Y and (**c**) X directions. (**d**) Cross-sectional micrographs of the hollow channels produced by chemically etching the inscribed sample in the last column of (c). Scale bar: 25 μm.

To examine the fabrication feature sizes, offered by loosely focusing the picosecond laser pulses into fused silica as a function of focal position along the propagation direction, we first inscribed multiple lines as schematically illustrated in Figure 3a. The lines, which are inscribed at different scan speeds and depths within fused silica, were organized into two grid arrays oriented in X and Y directions, as shown in Figure 3b,c, respectively. The scan speed and the depth of the focal position can be identified for each inscribed line, as indicated in Figure 3b,c. The average laser power was fixed at 1.65 W in the writing of the lines in both of the two arrays, corresponding to an intensity [20] of $3.13\times 10^{12}\;W/{\mathrm{cm}}^{2}$, and the laser pulses were always polarized along the Y direction. The results in Figure 3 were obtained using laser pulses negatively chirped to 10 ps. However, as shown in Figure 1, the same results can also be obtained if one chooses to use positively chirped pulses as long as the pulse duration is properly chosen. 

We noticed that the cross section of all the inscribed lines showed a similar geometry of a nearly circular shape, which are insensitive to scan speed, depth of focal position, as well as the direction of laser writing. The difference is that with an increasing scan speed, the color in the cross section captured under the microscope in a reflective mode becomes lighter, indicating that a weaker modification of fused silica will be generated with the decrease of the irradiation dose at the increasing scan speed. Since, in the 3D glass printing with the FLICE, a chemical wet etching must be carried out for selective removal of the modified fused silica by the laser pulses, it is important to examine the cross section of hollow channels produced after the chemical wet etching in KOH solution, as shown in Figure 3d. The lateral and axial feature sizes revealed by all the cross-sectional micrographs in Figure 3d are ~20 μm, despite the fact that the lines are inscribed at various depths across a range of 5 cm. This means that the fabrication can maintain an unchanged feature size from the bottom to the top of the glass without the additional need to compensate the aberration originated from the refractive index mismatch between air and glass. A high scan speed of 40 mm/s is chosen for inscribing the lines in Figure 3d, indicating that the depth-insensitive focusing of picosecond pulses is suitable for high throughput manufacturing of 3D objects in glass.

Another unexpected observation in the FLICE with laser pulses of ~10 ps is that nanograting formation can be avoided, as we have discussed in another recent publication [21]. It is well known that under the femtosecond laser irradiation, nanogratings tend to form inside various transparent materials, including glass materials such as fused silica as well as several crystals [22,23]. The mechanism is still under debate as this effect has been identified to play significant roles in the applications of microfluidics and photonics [24,25]. Nevertheless, the nanograting leads to sensitive etching selectivity depending on the orientation of the polarization of the writing laser beam, which increases the complexity of the beam steering system due to the requirement on the dynamic control of the polarization orientation in the 3D glass printing. Because we can eliminate the formation of nanogratings in fused silica with the picosecond pulses whilst still maintain the highly selective etching as shown in Ref. 21, we are able to perform 3D laser printing without the need for manipulating the polarization of the writing laser beam in real-time. This greatly simplifies the beam steering in the printing system, making the whole printing process more robust and easier to put into practice. 

### 3.2. Optimization of the Slice Thickness and Characterization of the Surface Quality

**Figure 4:**
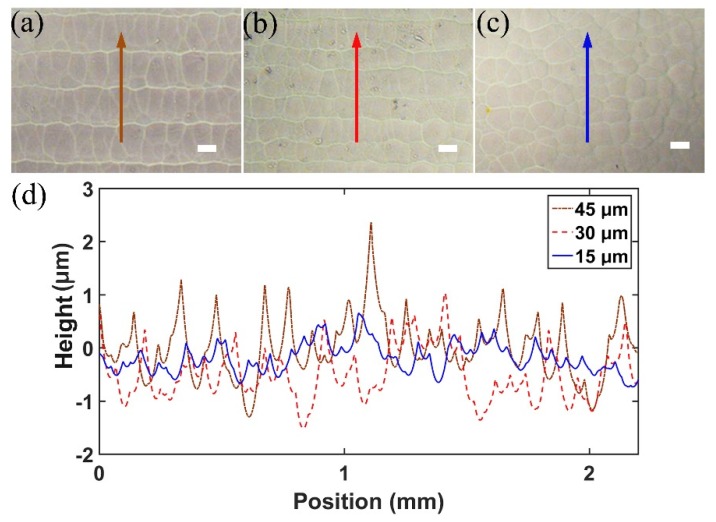
Surface morphologies measured on samples written with the slice thicknesses set at (**a**) 45 μm; (**b**) 30 μm; and (**c**) 15 μm. (**d**) The measured 1D surface profiles of the samples in (a), (b), and (c) are shown by the brown dotted, red dashed, and blue solid lines, respectively. Scale bar: 25 μm.

It is shown in Figure 3 that an isotropic feature size on the level of ~20 μm, which is independent of the depth of the focal position, can be obtained by loosely focusing the picosecond laser pulses into fused silica. However, for fabricating the 3D objects, chemical wet etching must be used, which leads to degradation of the fabrication resolution. To determine the limit on the fabrication resolution along the longitudinal direction, we scanned the focused picosecond laser pulses in the glass with different slice thicknesses and etched the samples in KOH to reveal the surface morphologies. As shown in Figure 4a–c, the surfaces produced with the slice thicknesses set at 45 μm and 30 μm show a clear laminar feature, owing to the fact that the thickness of each modification layer is less than both slice thicknesses. When the slice thickness is reduced to 15 μm, the laminar feature disappears, leaving behind a uniform surface with a typical morphology given rise to by the FLICE. It is also shown in the 1D surface profiles of the samples measured with a surface profiler, as shown in Figure 4d, that periodic ripples oriented along the horizontal direction, with a height between 1 μm and 2 μm and a height around 1 μm, appear for the surfaces produced at the slice thicknesses of 45 μm and 30 μm, which agrees well with the observations in Figure 4a,b. However, when the slice thickness is reduced to 15 μm, the 1D profile along the cutting lines in Figure 4d only shows random peaks without a significant periodicity. 

**Figure 5:**
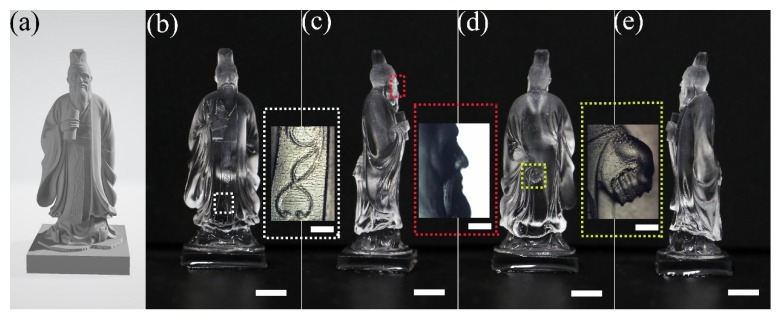
A laser printed sculpture of Confucius in fused silica. (**a**) The model and the (**b**) front, (**c**) left, (**d**) back, and (**e**) right sides of the sculpture. The details of the decorative pattern on the cloth, the right side of his face, and the left hand hanging behind his body are shown in the insets on the right-hand side of the images in (b), (c) and (d), respectively. Scale bar at the bottom: 5 mm. Scale bar in insets: 1 mm.

In principle, the results in Figure 4 indicate that a longitudinal feature size between 15 μm and 30 μm had been achieved with the picosecond laser 3D printing in glass, which agrees with the ~20 μm feature size as shown in Figure 3d. Unfortunately, there is a physical limit so far in choosing such a small slice thickness for printing large 3D structures in thick fused silica. The problem is that when ultrafast laser pulses are focused into glass, the modified material will have a volume expansion which builds up stress inside the glass. For large-scale internal modifications, the stress will be high and can give rise to multiple cracks which spoil the printed 3D objects. To solve this issue, we need to reduce the filling ratio in performing laser writing. This results in a lower fabrication resolution than that allowed by the voxel size of 3D laser writing. In the current experiment, the slice thickness is finally set at 50 μm to enable 3D printing of objects with heights up to 3.8 cm, as shown in Figure 5. 

### 3.3. 3D Printing of Macro-Scale Objects in Glass

The optimum inscription condition determined in Figure 3 allows us to print macro-scale 3D objects at micro-scale feature sizes. Figure 5 shows a 3.8 cm high sculpture, which is a statue of Confucius. The model of the sculpture is shown in Figure 5a, and the printed sculpture is presented from different angles of view in Figure 5b–e in the order of (b) front, (c) left, (d) back, and (e) right sides. The details of the decorative pattern on the cloth, the right side of his face, and the left hand hanging behind his body are shown in the insets on the right-hand side of the images in Figure 5b–d, respectively. It proves that the entire sculpture is printed with a decent fabrication feature size from top to bottom. The surface of the whole sculpture appears smooth, although it does not reach the level of the mirror-like surface quality typically produced with mechanical polishing. Improvement on the surface quality is possible with elaborated post-annealing or CO_2_ laser polishing, which requires much effort to optimize and will be investigated in the future. 

**Figure 6:**
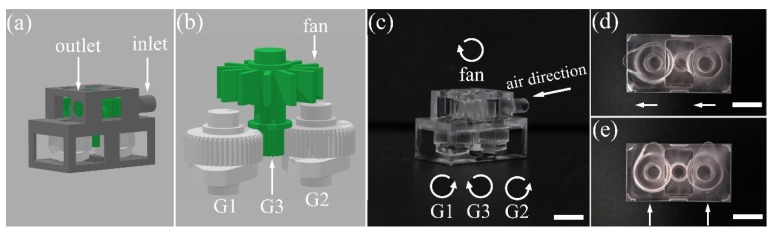
A laser printed air turbine in fused silica. (**a**) The whole air turbine model. Inlet and outlet for air injection are indicated. (**b**) The interior of the turbine including a turbine fan, a driving gear (G3) and two driven gears (G1 and G2). Each of G1 and G2 is connected with a cam. (**c**) Digital-camera captured image of the fabricated turbine. The air direction and rotation direction of the fan, as well as the rotation directions of G1, G2 and G3 from a top view are all indicated by the curved arrows in (c). (**d**) The initial position of the two cams is pointing to the left as indicated by the two arrows. (**e**) Both the cams are rotated in a clockwise direction by 90° as a result of the injected airflow. Scale bar: 5 mm.

Besides, we demonstrated an air turbine with movable parts directly fabricated within glass without any assembling process. The model of the air turbine is illustrated in Figure 6a,b. As shown by Figure 6b, the micromachine is composed of an air fan, one driving gear and two driven gears, as well as two cams. The driving gear is fabricated with the turbine fan as an integral component to ensure a robust physical connection between the gear and the turbine fan. The two driven gears can be wound by the driving gear when airflow drives the fan to rotate. Each of the driven gears is connected with a cam. The laser printed air turbine is presented in Figure 6c. The micro-machine is functional as we can rotate the cams by injecting airflow from the inlet. The synchronized motion of the two cams is evidenced by the images in Figure 6d,e, in which the orientations of the cams are changed as indicated by the white arrows. Thanks to the capability of fabricating large objects at high resolution, the demonstrated technique offers potential for manufacturing precision instruments, tools and machines in various research and application fields.

## 4. Conclusions

To conclude, we have demonstrated 3D laser printing of glass-based macro-scale objects with heights up to ~3.8 cm at a feature size of a few tens of micrometers. With the scan speed reaching 40 mm/s and the layer spacing being set at 50 μm, as we demonstrated in the current experiments, the fabrication efficiency can be determined to be 0.16 mm^3^/s or 38200 voxels/s [26]. Further improvement on the printing efficiency will be done in the near future by combining a 2D galvo scanner with the 2D motion stage. This design will allow both a high printing speed and a large printing area. The novel 3D glass printing technique is established based on two unconventional characteristics in the interaction of loosely focused picosecond laser pulses with fused silica, namely, the depth-independent focusing and the elimination of the self-organized nanograting. The physical mechanisms behind these interesting effects have not yet been clarified. We stress that the interaction of ultrafast laser pulses with transparent media under loose focusing condition is a largely unexplored area of research, which shall inspire significant interest for further investigations. The high-resolution 3D printing of macro-scale objects in glass is expected to have implications in the fields of photonics, microfluidics, and high-precision mechanics.
